# Assessment of the Authenticity of Whisky Samples Based on the Multi-Elemental and Multivariate Analysis

**DOI:** 10.3390/foods11182810

**Published:** 2022-09-12

**Authors:** Magdalena Gajek, Aleksandra Pawlaczyk, Elżbieta Maćkiewicz, Jadwiga Albińska, Piotr Wysocki, Krzysztof Jóźwik, Małgorzata Iwona Szynkowska-Jóźwik

**Affiliations:** 1 Faculty of Chemistry, Institute of General and Ecological Chemistry, Lodz University of Technology, Zeromskiego 116, 90-924 Lodz, Poland; 2 Faculty of Mechanical Engineering, Institute of Turbomachinery, Lodz University of Technology, Wolczanska 219/223, 90-924 Lodz, Poland

**Keywords:** authentication, adulteration, fake, whisky, elemental analysis, ICP-MS, ICP-OES, CVAAS, spirits, principal component analysis, alcohol aging, isotope ratios

## Abstract

Two hundred and five samples of whisky, including 170 authentic and 35 fake products, were analyzed in terms of their elemental profiles in order to distinguish them according to the parameter of their authenticity. The study of 31 elements (Ag, Al, B, Ba, Be, Bi, Cd, Co, Cr, Cu, Li, Mn, Mo, Ni, Pb, Sb, Sn, Sr, Te, Tl, U, V, Ca, Fe, K, Mg, P, S, Ti and Zn) was performed using the Inductively Coupled Plasma Mass Spectrometry (ICP-MS), Inductively Coupled Plasma Optical Emission Spectrometry (ICP-OES) and Cold Vapor-Atomic Absorption (CVAAS) techniques. Additionally, the pH values of all samples were determined by pH-meter, and their isotopic ratios of ^88^Sr/^86^Sr, ^84^Sr/^86^Sr, ^87^Sr/^86^Sr and ^63^Cu/^65^Cu were assessed, based on the number of counts by ICP-MS. As a result of conducted research, elements, such as Mn, K, P and S, were identified as markers of whisky adulteration related to the age of alcohol. The concentrations of manganese, potassium and phosphorus were significantly lower in the fake samples (which were not aged, or the aging period was much shorter than legally required), compared to the original samples (in all cases subjected to the aging process). The observed differences were related to the migration of these elements from wooden barrels to the alcohol contained in them. On the other hand, the sulfur concentration in the processed samples was much higher in the counterfeit samples than in the authentic ones. The total sulfur content, such as that of alkyl sulfides, decreases in alcohol with aging in the barrels. Furthermore, counterfeit samples can be of variable origin and composition, so they cannot be characterized as one group with identical or comparable features. Repeatedly, the element of randomness dominates in the production of these kinds of alcohols. However, as indicated in this work, the extensive elemental analysis supported by statistical tools can be helpful, especially in the context of detecting age-related adulteration of whisky. The results presented in this paper are the final part of a comprehensive study on the influence of selected factors on the elemental composition of whisky.

## 1. Introduction

Extremely fast development of trade and international exchange of products and food mobility brought an unprecedented variety of food products to consumers. However, nowadays, consumer awareness regarding the quality and authenticity of the food they buy and consume was raised significantly. Moreover, a study conducted over a decade ago indicated that as many as 82% of the customers considered geographical origin as a quality indicator before purchasing food products [1]. Literature reports clearly suggest that numerous cases of food adulteration have been reported, including the use of substances that pose a threat to the health and life of consumers. Examples of such activities can be given as follows: mixing melamine and wheat gluten to increase the protein content [2], contamination of paprika powder with lead oxide [3], addition of red lead (Pb_3_O_4_) to cayenne pepper to achieve a vibrant color [4]. In turn, honeys are often adulterated to increase their shelf-life and nutritional value, by adding glucose–fructose syrups, corn syrups, invert sugar syrups or by admixing with imported honeys of poorer quality [5,6]. Thus, food authenticity is an important matter in the case of quality control and assurance of food safety. The authentication of food concerns many aspects, including misleading about origin, mislabeling and adulteration, which is defined as a process by which the quality or the nature of a given product is reduced due to the addition of a foreign or an inferior substance and removing a vital element [7,8].

The need for precise and valid analytical techniques for food investigations is increasing because of the continuously rising food deception around the world [9,10,11]. Fortunately, a range of potential analytical techniques for the authenticity termination and traceability of food products is extensive. Among them, the following methods can be distinguished: spectroscopic techniques [12,13,14,15] (including those based on isotopic ratios [16,17]), separation techniques [6,18], neutron and proton-based nuclear techniques [19], as well as advanced DNA-based techniques [10,20]. Elemental analysis has long been used in research connected with food authenticity, including discrimination of geographical origin [7], organic versus conventional cultivation [21] or free range to compare with conventionally farmed products [22]. Numerous literature reports indicate that elemental fingerprinting also proved its usefulness for the differentiation of origin of wine [15,23], olive oil [24], honey [6,25], coffee [26], tea [27], cheese [28], vegetables and fruits [29] and also spices and food additives [30]. Food products consist of numerous compounds, including carbohydrates, peptides, lipids, fatty acids, amino acids, organic acids, nucleic acids and other small molecules (aromas, dyes, preservatives and other exogenous compounds) [31]. Due to the complexity of the ingredients in the food, using chromatographic methods it makes possible to obtain unique molecular fingerprints, which has a huge potential in differentiation during the authentication process [30]. Separation techniques were used for food authentication and geographic identification of the following: apple juice [32], kiwifruit juices [33], wine [34], honey [6], saffron [35], tomatoes [36], ginger [37], whisky [38,39,40,41,42] and fruit spirits [43]. Moreover, the isotopic ratios were successfully used in food authentication because stable isotope ratios are dependent on the climatic and soil conditions, as well as geographical origin of food ingredients [30]. The isotope ratios mostly investigated in food authentication are ^2^H/^1^H, ^13^C/^12^C, ^15^N/^14^N, ^18^O/^16^O, ^34^S/^32^S, ^84^Sr/^86^Sr, ^87^Sr/^86^Sr, ^88^Sr/^86^Sr ^206^Pb/^204^Pb, ^207^Pb/^204^Pb and ^208^Pb/^204^Pb [44,45]. Literature reports indicate that techniques based on the measurement of isotope ratios are most often used for authentication of cheeses [46], sweet cherries [47], lentils [48] bell pepper [49], wheat [50], wine [51,52] and vodka [53].

Due to the great popularity and high price, premium whisky is one of the most frequently counterfeited alcoholic beverages. The process of counterfeiting whisky usually involves blending a cheaper version of whisky belonging to the same category as the genuine brand, mixing a cheap local alcohol with the original brand of whisky or using a cheap local alcohol with added flavorings and coloring as a genuine product [54]. Another possibility of counterfeits in the case of whisky is the use of a different type of barrel, as well as a much shorter aging period compared to the manufacturer’s declarations. The most important quality characteristics, particularly in the case of premium brands, are the maturation period and the history of the casks in which whisky was matured. Thus, during the authentication process of whisky, a number of facts have to be taken into consideration. The water, the cereals, the use of peat smoke during grain malting and the equipment applied in the distillation process will have an influence, to a greater or lesser extent, on the final product. During the aging of the raw distillate in the barrel, significant changes take place in the chemical composition of the alcohol, which results in the “softening” of the product [42,55]. As previously noted, the analytical techniques most commonly used to authenticate and identify the geographical origin of whisky are chromatographic methods [38,39,40,41,42]. They allow finding characteristic compounds and determine aroma profiles, which can then be used to define the quality and authenticity of the tested whisky [56]. Especially the analysis of esters, which have the greatest impact on the aroma of the alcohol, enables an assessment of the aging process and, as a result, the verification of the authenticity of the age of whisky [57,58].

Taking into account the number of scientific studies dealing with the authentication and identification of the origin of food products, most of articles refer to wines; then fruit, vegetables and cereals; and, finally, meats, oils and fats. The available scientific data show that less than 10% of all publications devoted to food authentication concern the analysis of beverages (including spirit, beers, soft drinks and mineral waters) [30]. To the authors’ knowledge, very few papers on metal analysis in whisky are available [59,60,61,62]. However, the use of the elemental profile to establish authenticity and provenance is extremely rare in the literature [60]. In the first part of the scientific study (The Elemental Fingerprints of Different Types of Whisky as Determined by ICP-OES and ICP-MS Techniques in Relation to Their Type, Age, and Origin [61]), the extensive elemental characterization of whisky samples was performed, including distinguishing alcohol samples based on their origin, type and age using statistical analysis and chemometric tests. The authors in this paper have not discussed the issues related to the authenticity of products or its possible identification. 

The main purpose of this work was to assess the authenticity parameter based on an extensive elemental analysis supported by appropriate statistical and chemometric tests. It should be emphasized that in this study wide range of measurements were carried out with the use of 3 analytical techniques (ICP-MS, ICP-OES and CV-AAS) to determine the concentrations of 31 elements in 205 whisky samples (170 authentic and 35 fake samples). Additionally, the pH value was measured for each of the analyzed alcohol samples, and the collected semi-quantitative data were used to determine the isotope ratios.

## 2. Materials and Methods

### 2.1. Samples

In this study, a total of 205 whisky samples were analyzed, including 170 samples of original products, which were discussed in the first part of the publication (The Elemental Fingerprints of Different Types of Whisky as Determined by ICP-OES and ICP-MS Techniques in Relation to Their Type, Age, and Origin [63]), as well as 35 samples of unidentified identity, called fake products, which were used as a reference group for the authenticity studies. Among the 35 samples, 9 different sources of their origin can be distinguished. The source of origin is understood to mean the producer or the place where the product was manufactured. These alcohols were distributed on various scales as analog of whisky products. To the authors’ knowledge, fake alcohols were not matured in wooden barrels or this stage was significantly reduced. However, the counterfeits whisky products were from sources that remain anonymous. The analysis was performed using the ICP-MS, ICP-OES and CVAAS techniques.

The information about whisky products categories was coded, and the manufacturers’ names are not given in this paper. Basic characteristics of the tested samples are included in Table 1.

### 2.2. Samples Preparation and Equipment

- ICP-OES, ICP-MS and CV-AAS

The sample preparation procedures and the measurement conditions are described in detail in the publication Elemental Fingerprint of Different Types of Whisky Determined by ICP-OES and ICP-MS techniques in Relation to Their Type, Age and Origin [61] and in our preliminary study (Multielemental Analysis of Various Kinds of Whisky [63]). Moreover, all validation procedures were analogous to those described in the first part of the paper.

- pH-Metr

Basic 20^+^ pH-meter (CARISON INSTRUMENTS S.A., Barcelona, Spain) was used to measure the pH values of the tested whisky samples. The pH-meter consists of a magnetic stirrer with automatic temperature stabilization and a combined electrode with glass and a silver chloride electrode placed in one holder. Before the measurement, the necessary calibration process was performed using buffers at pH 4.01, 7.00 and 9.21 (HACH Company, Düsseldorf, Germany). Measurements were carried out during a three-day analytical cycle. Three replicates were performed for each sample, and the average result was taken as the final result. After analyzing 20 samples, calibration was repeated.

### 2.3. Data Analysis

The STATISTICA 12.5 (New York, NY, USA) software was employed for raw data processing. The first step was to check the normality of the distribution of the studied variables. In this order, Kołmogorow–Smirnow tests were applied. On the basis of the tests, the hypothesis of normal distribution was rejected for all studied elements and isotope ratios, as well as pH-value (for the significance level α = 0.05). Then, the existence of statistically significant differences was checked. For this purpose, the Kruskal–Wallis non-parametric test was used. In the final phase, data were investigated by multivariate chemometric analysis. To increase the interpretability of the results, principal component analysis (PCA) was applied. 

## 3. Results and Discussion

### 3.1. Level of Metals in Analyzed Whisky Samples

In this study, the concentration of 31 elements in 205 whisky samples and products of unknown identity was determined. A total of 170 samples are authentic products, the concentrations of which were listed in the first part. The remaining 35 items are false objects and the obtained results for this group regarding their elemental profile were given in this paper. The ICP-MS technique was used to determine the concentration of the following elements: Ag, Al, B, Ba, Be, Bi, Cd, Co, Cr, Cu, Li, Mn, Mo, Ni, Pb, Sb, Sn, Sr, Te, Tl, U and V, while elements, such as Ca, Fe, K, Mg, P, S, Ti and Zn, were measured with the ICP-OES technique. The CVAAS technique was used to determine the total mercury content.

In terms of 35 samples of counterfeit products, some of the obtained results were below the quantification limits. The Hg concentration was below the limit of quantification in each case. Te was not determined in 31 samples. Ag was not determined in 19 samples, P in 15 and Fe in 13. Sb and Bi were not detected in 12 samples, while Cd and Ti in 9 samples. Zn was not found in six samples; Mo and Tl in four; and Al, V, Sn and Pb in three samples. U was not identified in two independent samples, while Li, Be and B were not quantified in one sample. 

In the first part of the publication, the basic statistical parameters of authentic products (170 samples) were summarized. Therefore, in Table 2 the same type of the information was given, such as the mean, median, minimum and maximum, but for the group of counterfeit products (35 samples). In each case, due to the rejection of the hypothesis of normal distribution, in order to assess statistically significant differences between the groups under consideration, the non-parametric Kruskal–Wallis test was applied.

The average contents of median values for the elements in the alcohol samples of unidentified origin decreased in the following order: Ca > K > S > B > Mg > Zn > Ba > Al > Cr > Sr > P > Fe > Cu > Ni > Mn > Ti > Li > Sn > Pb > Co > Mo > V > Cd > Bi > Sb > U > Be > Tl > Ag > Te > Hg. The order of elements for authentic samples was similar with the general trend from macro to micro elements. However, it should be noted that in the case of original products, elements, such as P and Cu, are listed higher in this order, while S lower than the presented order for non-original samples.

The authors of this paper referred to the internal national standards that define the maximum permissible content of selected metals (Cd, Pb) in high-percentage alcohols [64], which were presented in the first part of the manuscript, decided also to check potential exceedances of heavy metals (Cd and Pb) in fake whisky samples. In the mentioned standards, the maximum lead content was set at 0.3 mg/L, and the cadmium one at 0.03 mg/L. This time, there were only exceedances in the case of cadmium. The exceedances of the maximum allowable concentrations concerned three samples (F10, F11 and F12), which came from a common source. The values recorded for Cd in these cases ranged from 32.25–65.90 µg/L.

### 3.2. Comparison of Elemental Profiles of Authentic and Counterfeit Whisky

In this experiment, a set of counterfeit and authentic samples was analyzed to reveal the possible differences between them, as well as to detect and identify the elemental fingerprint group of genuine and fake whisky. Apart from the above-mentioned 30 elements (Hg was omitted because its concentration in each sample was below the limit of quantification) and the pH value, in the analysis, the values of Sr and Cu isotope ratios were also used. These ratios were calculated based on the number of counts for each of the isotope as a result of the semi-quantitative analysis. In the case of Sr isotopes, the interference from Rb was corrected. For copper, an analysis was performed on the basis of the ^63^Cu/^65^Cu isotope ratio. In turn, for Sr, the following isotopic ratios were used: ^88^Sr/^86^Sr, ^84^Sr/^86^Sr, ^87^Sr/^86^Sr, as these are the parameters most frequently used in food authentication [45]. 

On the basis of the Kruskal–Wallis test, the existence of statistically significant differences in the concentration of the following elements was demonstrated: Be, Ca, Cu, Li, Mg, Mo, S, Sn, Sr and pH value (Table 3). In all mentioned cases the level of significance (*p*) was less than 0.05. 

Comparing the median values of the two groups under consideration (fake and authentic whisky samples) in each case, except for copper, higher values were noted for products with unidentified identity. Although the highest content of copper was recorded in the fake sample (33.21 µg/L), the median and mean values of the samples belonging to the group of authentic products were much higher. However, it should be noted that in the group of false samples there were five objects with a much higher concentration of copper. These were samples coded as F4 and F5 and from F9 to F11 with a copper content in the range from 12.89 to 33.21 µg/L. As emphasized in the first part of the work, the presence of copper in alcohol is undoubtedly related to the material of the apparatus used in the production process, and more specifically during distillation. Therefore, the alcohols coded as F4, F5, F9, F10 and F11 have most certainly been distilled in copper stills, resembling the high-quality single malt whisky. As it was underlined in the previous paper, differentiation of the authentic samples may be influenced by several overlapping parameters. Moreover, counterfeit samples can be of variable origin and composition, so it is impossible to characterize them as one group with identical or comparable attributes. When the influence of overlapping parameters was eliminated, in the case of authentic samples, the increasing concentration of V, Cr, Ni, Sr, Sb, Bi, Zn, Mg, K and P with the age of the analyzed samples was revealed (despite the lack of statistically significant differences). A similar result was recorded for the comparison of authentic and false objects in this study. Despite the lack of statistically significant differences, higher values of both the median and mean of Mn and P and the median value for K were recorded for the genuine samples, which were maturated (minimum 3 years). Thus, it is possible to clearly indicate the influence of aging on the levels of phosphorus and manganese and potassium, as these elements can be selected as markers for the identification of products with adulterated maturation. The chemical composition of wood is the explanation for the higher content of the above-mentioned elements in the authentic samples in relation to the false ones. Unadulterated whisky is matured in oak barrels, usually incinerated from the inside. The presence of phosphorus and potassium is directly related to the oxides formed during the firing of wooden barrels for aging alcohol. On the other hand, phosphorus, as a macroelement necessary for plant development, may accumulate in various parts of plants when migrating from the soil. The main form of phosphorus in soil is phosphates, including manganese phosphates [65,66]. In addition, manganese compounds are used as wood preservatives, which may also affect the content of this element in alcohol stored in oak barrels [67]. Thus, the longer the alcohol stays in contact with wooden barrels, the greater the migration of these elements into the product. It is true that the aforementioned average concentration of copper was higher in authentic samples, i.e., those subjected to the aging process, however, the content of this element should be associated with the equipment used for production rather than with the age parameter. 

Among the elements listed in Table 3, for which the existence of statistically significant differences has been demonstrated, the presence of sulfur should be commented on. As reported in the literature data, sulfur volatile compounds generated during the whisky production process influence their quality to a large degree [68]. The selected alkyl sulfides (dimethyl sulfide (DMS), dimethyl disulfide (DMDS) and dimethyl trisulfide (DMTS)) have been recognized as age markers for whisky, as the level decreases with the time the alcohol spends in the barrel [69,70]. Comparing the mean and the median values of the groups of false and authentic samples, it is clear that the concentration of S in the set of counterfeit samples (not subjected to aging or with a falsified aging period) was an order of magnitude higher than in the original ones (which in each case were samples aged by at least 3 years). Thus, both the concentration of sulfur compounds, as evidenced by the literature, and the total sulfur content, as shown in this study, decrease with the aging of alcohol.

Also, the much higher pH value in the case of fake samples, as compared to the authentic ones, is worth emphasizing. This applies to both the mean and the median values. Although the set of authentic samples is much more numerous than the samples of unidentified identity, the pH values obtained in this group were much more similar and were in the acidic pH range. The counterfeit alcohol samples, on the other hand, had the pH ranging from 2.79 to 8.70, i.e., from acid to alkaline. Adherence to strict standards in the whisky production process ensures that certain physical and chemical parameters of alcohol are maintained within a given brand, including the characteristic pH value of the product. The large discrepancy in the results of the pH value in a small group of fake samples (including samples from a common source) suggests a lack of compliance with production standards and certain randomness during the production of this type of alcohol.

The comparison of the Cu and Sr isotope ratios of the genuine and false sample groups did not provide significant information allowing their better differentiation.

In the next step, the projection of cases on the factor plane for reduced data set was made. Since the significant influence of aging on the elemental profile of whisky had already been proven in earlier work, the age parameter was eliminated. Therefore, during the comparison of false and genuine samples, only the original samples were taken into account, which were aged for the legally required period (3 years).

**Figure 1:**
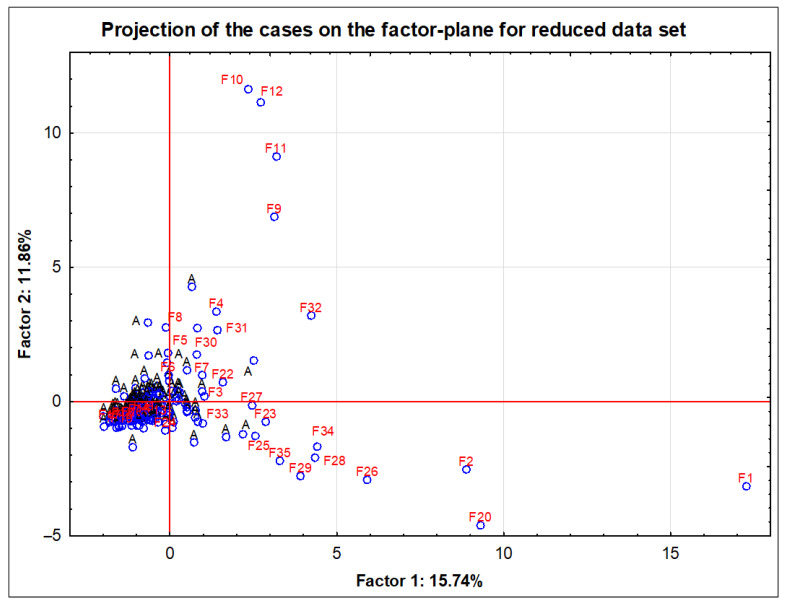
PCA score plot of 3-year-old authentic (A) and fake (F1–35) whisky samples.

As shown in Figure 1, quite a good separation between genuine and counterfeit samples using PCA was achieved. The vast majority of authentic samples are accumulated in one area of the graph (around the point of intersection of the coordinate axes), while the points belonging to the false samples are scattered over throughout the plot. This area contains over 70% of alcohol samples with unidentified identity. Despite the much smaller number of counterfeit samples, their large diversity in composition makes it impossible to characterize them as one group with similar physicochemical characteristics. Repeatedly, other authors have indicated that it is extremely difficult to find a marker occurring only in fake samples [42,71,72,73,74,75]. Most often, the problem arises from the type and nature of the adulterations. Depending on whether the adulteration concerns a lower alcohol content than the standard required [73] or on the addition of esters, aldehydes or organic acids [71,72], in order to reflect the age, taste, smell and quality of a given brand, a different and individual approach should be taken. Nonetheless, under such conditions, nontargeted screening followed by chemometric analysis can be a powerful instrument to uncover deviations from typical authentic whisky fingerprints.

Figure 1 resembles an analogous projection presented in the work of Stupak et al. [42]. The authors of the aforementioned work separated the samples of counterfeit and original whisky on the basis of selected markers measured with chromatographic techniques. In this case, in the PCA plot, all points belonging to the group of genuine products (both single malt and blended) were clustered in one common area, while objects belonging to the fake samples are dispersed across the graph.

### 3.3. Counterfeit Whisky Analysis

**Figure 2:**
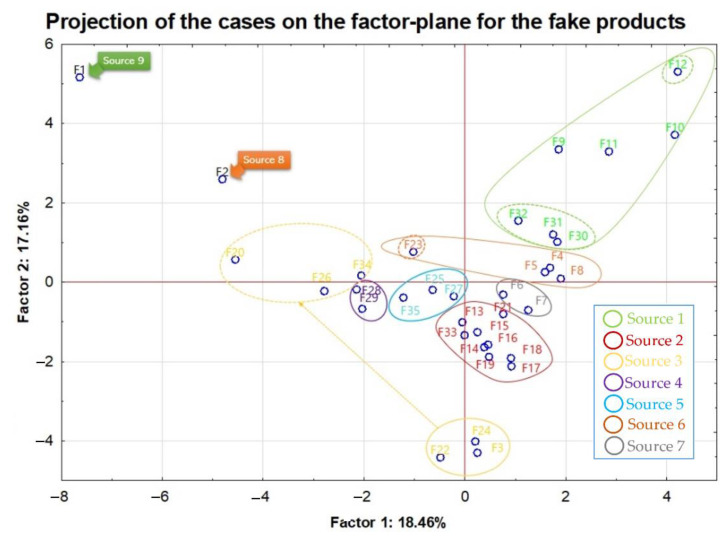
PCA score plot of fake whisky with division on 9 different sources of samples.

In the next steps, only samples marked as fake (35) were discussed separately with division to their sources of origin (1–9). On the basis of the Kruskal–Wallis test, the existence of statistically significant differences in the concentration of the following elements was demonstrated: B, Bi, Cd, Co, Fe, Mn, Mo, Ni, Pb, Sb and Zn. In each case, the level of significance (*p*) was less than 0.05. The most important statistical information connected with the division of fake samples against the sources is included in Table 4. It is worth noting that statistically significant differences for each of the elements, every time concerned, the source of the counterfeit whisky samples was marked as the number 2 (indicated as red color on Figure 2). Moreover, taking into account the median value for all elements listed in Table 5 (except Sn), the lowest concentrations were recorded for source 2.

In the analyzed set of fake samples, nine different, independent sources were distinguished and according to this criterion a division was made and what is worth mentioning is the fact that within these separated groups, alcohol samples of a completely different nature were observed. This means that they were produced by one manufacturer, but some of them are “raw” products, i.e., distillates that have not undergone any treatment to change their color or taste, whereas others are finished products intended for sale and consumption. However, the tendency that can be noticed in the projection of the cases on the factor plane for the fake products presented in Figure 2 is the grouping of samples within a common source. Each group has been marked with a different color. Sources 8 (F2) and 9 (F1) are represented by single samples. Sample F1 (source 9) is distinguished by the highest values of Li, Mn, Sr and Ba in relation to the other counterfeit samples, hence its extreme position on the graph presented below. Within the source 1 (marked in green), a cluster of items from F30 to F32 can be distinguished. These are samples of the same alcohol coming probably from one production batch but taken from three independent bottles. It should be mentioned that this alcohol has been enriched with wood extracts in order to give it the characteristic whisky aromas. The other samples in this group are of a completely different nature. Moreover, samples F9–12 and F30–32 contain the highest concentrations of Cd in the tested set of false ones. For items F10–12, the permissible level of this element has been exceeded. The samples from sources 2, 4, 5 and 7 in Figure 2 form the most central, individual clusters. An interesting group is consisted of the samples from source 3 marked in yellow in Figure 2. Points F3, F22 and F24 are samples of high-strength distillates. In turn, samples F20, F26 and F34 are flavored products, which are made from these distillates. They have been enriched with sugar and fruit juices. These products were supposed to resemble whisky-based fruit liqueurs. 

## 4. Conclusions

Mn, K and P are elements with higher concentrations recorded in the case of authentic samples. Their presence is directly related to the aging period of alcohol and can be indicated as markers for the identification of fraudulent activity in this respect. Another indicator certainly associated to the whisky maturation process in barrels is S. In products that were not aged or the aging period was much shorter than legally required (fake samples), the concentration of this element was much higher, compared to the original samples (in all cases subjected to the aging process). Counterfeit samples can be of variable origin and composition, so they cannot be characterized as one group with identical or comparable attributes. Often, the element of randomness dominates in the production of such alcohols. The use of unsuitable ingredients or production equipment, as well as inadequate knowledge in this field, cause the lack of repeatability of the taste and smell characteristics of alcohol beverages. This is evidenced by, for example, the failure to meet the standards for the maximum content of heavy metals in high-percentage alcohols. The adulteration of food products, including whisky, may be of various characters. It can refer to a reduced percentage of alcohol or the addition of various organic compounds to improve the visual and flavor properties. Therefore, the identification of the falsification of a different nature requires the use of a wide range of analytical techniques and often an individual approach. 

The results presented in this article constitute the final part of a broad characteristic of the elemental composition carried out for 205 whisky samples. As our research revealed, the elemental analysis supported by statistical tools may provide beneficial information, especially in the context of the differentiation of alcohol samples in regard to such parameters as type, origin and detecting age-related adulteration of whisky.

## Figures and Tables

**Table 1:** Characteristics of the tested set of samples.

n	Authentic	Fake								
	170	35								
		Number of Samples from a Given Source								
		S1–7	S2–9	S3–6	S4–2	S5–3	S6–4	S7–2	S8–1	S9–1
Total	205									

S1–S9 code of source of origin (e.g., S1—source no 1).

**Table 2:** Basic statistics for determined elements for all counterfeit samples (n = 35) [µg/L].

Element	n	Mean	Median	Min	Max	Element	n	Mean	Median	Min	Max
Ag	35	1.280	<LOQ	<LOQ	8.600	Sb	35	0.540	0.300	<LOQ	3.000
Al		168.4	163.3	<LOQ	470.7	Sn		13.89	9.810	<LOQ	34.70
B		3794	2397	<LOQ	19.02	Sr		133.0	53.72	14.146	765.1
Ba		199.6	189.0	117.3	378.0	Te		0.060	<LOQ	<LOQ	1.100
Be		0.130	0.110	<LOQ	0.500	Tl		0.210	0.030	<LOQ	2.100
Bi		3.220	0.600	<LOQ	25.80	U		0.360	0.190	<LOQ	3.100
Cd		6.110	0.760	<LOQ	65.90	V		1.680	0.910	<LOQ	10.40
Co		9.920	5.260	1.409	42.20	Ca		35.73	22.91	1994	271.1
Cr		182.5	112.3	54.57	770.3	Fe		174.7	29.98	<LOQ	2735
Cu		2383	56.86	1.922	33.21	K		97.09	10.88	<LOQ	670.6
Li		67.12	19.25	<LOQ	825.4	Mg		5370	1577	465.4	33.07
Mn		76.75	51.39	2.377	438.7	P		7352	74.29	<LOQ	56.79
Mo		11.07	1.590	<LOQ	108.4	S		20.89	14.68	197.6	231.7
Ni		62.71	39.86	2.418	411.0	Ti		43.49	25.35	<LOQ	316.8
Pb		12.84	11.21	<LOQ	35.60	Zn		2987	274.8	<LOQ	39.82

**Table 3:** Contents of selected elements (with statistically significant differences) in the measured fake and authentic alcohol samples (n = 205) [µg/L].

Element	Code	N	Mean	Median	Min	Max	Std. Dev.
^9^Be	A	170	0.100	0.092	<LOQ	0.300	0.050
	F	35	0.130	0.120	<LOQ	0.500	0.100
^59^Co	A	170	4.530	2.468	0.406	74.90	7.870
	F	35	9.920	5.260	1.409	42.20	10.10
^63^Cu	A	170	473.7	216.0	16.25	5252	736.4
	F	35	4021	56.86	1.922	33,212	7367
^7^Li	A	170	21.36	12.27	0.474	399.5	35.40
	F	35	67.12	19.25	<LOQ	825.4	140.8
^95^Mo	A	170	1.790	1.066	<LOQ	32.30	3.320
	F	35	11.07	1.590	<LOQ	108.4	30.30
^60^Ni	A	170	24.01	12.96	3.201	301.3	33.68
	F	35	62.71	39.86	2.418	411.0	73.70
^118^Sn	A	170	9.800	4.672	<LOQ	44.50	11.31
	F	35	13.89	9.810	<LOQ	34.70	11.00
^88^Sr	A	170	47.18	45.81	15.84	119.2	19.80
	F	35	133.0	53.72	14.15	765.1	168.8
Ca 393.366	A	170	14.66	9185	723.8	175.4	17.98
	F	35	35.73	22.91	1994	271.1	50.19
Mg 279.553	A	170	1487	1046	208.5	11.55	13.93
	F	35	5370	1577	465.4	33.07	764
S 180.731	A	170	7126	4648	296.7	69.91	8654
	F	35	20.89	14.68	197.6	231.7	39.56
pH value	A	170	3.63	3.63	1.95	6.20	0.68
	F	35	4.71	4.39	2.79	8.70	1.50

**Table 4:** Groups with statistically significant differences.

Statistically Significant Differences	Elements
Source 6–Source 2	B
Source 3–Source 2	Fe; Mn; Mo; Sn
Source 1–Source 2	Bi; Cd; Co; Ni; Pb; Zn

**Table 5:** Contents of selected elements (with statistically significant differences) in the measured fake alcohol samples (n = 35) [µg/L].

Element	No. of Source	N	Mean	Median	Min	Max	Std. Dev.
^11^B	1	7	2064	2238	<LOQ	3289	1055
	2	9	1803	1704	190.7	3758	1158
	3	6	2503	2728	1704	3059	657.0
	6	4	9078	8413	5970	13.52	3318
^209^Bi	1	7	12.75	10.35	9.387	25.77	5.870
	2	9	<LOQ	<LOQ	<LOQ	<LOQ	<LOQ
	3	6	0.847	0.419	<LOQ	3.543	1.347
	6	4	2.300	2.705	<LOQ	3.790	1.618
^111^Cd	1	7	26.05	11.06	3.204	65.90	28.11
	2	9	0.019	<LOQ	<LOQ	0.128	0.042
	3	6	2.690	2.166	<LOQ	7.325	2.857
	6	4	0.724	0.659	0.171	1.410	0.510
^59^Co	1	7	26.09	23.90	13.77	42.21	9.000
	2	9	3.840	3.436	1.409	7.613	1.895
	3	6	7.218	5.584	3.698	12.53	4.056
	6	4	5.985	4.690	3.504	11.06	3.430
^55^Mn	1	7	39.55	37.18	16.85	73.94	18.72
	2	9	14.12	5.192	2.377	64.16	20.26
	3	6	143.4	81.27	64.16	438.7	147.1
	6	4	49.89	31.59	6.299	130.0	58.44
^95^Mo	1	7	1.280	1.560	<LOQ	2.130	0.840
	2	9	0.289	0.242	<LOQ	0.988	0.339
	3	6	56.68	59.58	1.982	108.4	56.76
	6	4	2.953	2.424	1.218	5.750	1.947
^60^Ni	1	7	79.75	57.46	34.79	136.2	43.85
	2	9	13.77	10.21	2.419	30.11	10.04
	3	6	110.4	47.66	19.14	411.0	150.9
	6	4	75.36	74.30	69.34	83.49	6.114
^208^Pb	1	7	29.65	30.75	22.99	35.60	4.430
	2	9	3.677	1.186	<LOQ	21.89	7.008
	3	6	10.72	13.38	3.569	13.42	4.308
	6	4	12.09	12.43	6.553	16.95	5.358
^118^Sn	1	7	4.330	4.737	<LOQ	8.600	2.860
	2	9	17.09	19.97	9.310	20.41	4.627
	3	6	29.63	29.59	23.42	34.65	3.617
	6	4	8.257	4.651	<LOQ	23.73	10.55
Fe 238.204	1	7	49.00	47.75	<LOQ	90.67	33.67
	2	9	0.036	<LOQ	<LOQ	0.316	0.105
	3	6	669.7	233.2	<LOQ	2735	1035
	6	4	7.496	<LOQ	<LOQ	29.98	14.99
Zn 213.856	1	7	11.01	5668	4353	39.82	12.80
	2	9	90.47	0.144	<LOQ	429.3	152.5
	3	6	859.7	725.1	111.2	1891	704.8
	6	4	4189	77.29	<LOQ	16.60	8276

<LOQ—limit of quantification

## Data Availability

The date are available from the corresponding author.
